# Development and Validation of a Tumor Mutation Burden–Related Immune Prognostic Model for Lower-Grade Glioma

**DOI:** 10.3389/fonc.2020.01409

**Published:** 2020-08-20

**Authors:** Wen Yin, Xingjun Jiang, Jun Tan, Zhaoqi Xin, Quanwei Zhou, Chaohong Zhan, Xianyong Fu, Zhaoping Wu, Youwei Guo, Zhipeng Jiang, Caiping Ren, Guihua Tang

**Affiliations:** ^1^Department of Neurosurgery, Xiangya Hospital of Central South University, Changsha, China; ^2^The Key Laboratory of Carcinogenesis of the Chinese Ministry of Health, The Key Laboratory of Carcinogenesis and Cancer Invasion of the Chinese Ministry of Education, Xiangya Hospital, Central South University, Changsha, China; ^3^Cancer Research Institute, Collaborative Innovation Center for Cancer Medicine, School of Basic Medical Science, Central South University, Changsha, China; ^4^Department of Clinical Laboratory, Hunan Provincial People's Hospital (First Affiliated Hospital of Hunan Normal University), Changsha, China

**Keywords:** tumor mutation burden (TMB), lower-grade glioma (LGG), immune infiltration, immune-related risk score system, nomogram

## Abstract

Tumor mutation burden (TMB) is a useful biomarker to predict prognosis and the efficacy of immune checkpoint inhibitors (ICIs). In this study, we aimed to explore the prognostic value of TMB and the potential association between TMB and immune infiltration in lower-grade gliomas (LGGs). Somatic mutation and RNA-sequencing (RNA-seq) data were downloaded from the Cancer Genome Atlas (TCGA) database. TMB was calculated and patients were divided into high- and low-TMB groups. After performing differential analysis between high- and low-risk groups, we identified six hub TMB and immune-related genes that were correlated with overall survival in LGGs. Then, Gene Set Enrichment Analysis was performed to screen significantly enriched GO terms between the two groups. Moreover, an immune-related risk score system was developed by LASSO Cox analysis based on the six hub genes and was validated with the Chinese Glioma Genome Atlas dataset. Using the TIMER database, we further systematically analyzed the relationships between mutants of the six hub genes and immune infiltration levels, as well as the relationships between the immune-related risk score system and the immune microenvironment in LGGs. The results showed that TMB was negatively correlated with OS and high TMB might inhibit immune infiltration in LGGs. Furthermore, the risk score system could effectively stratify patients into low- and high-risk groups in both the training and validation datasets. Multivariate Cox analysis demonstrated that TMB was not an independent prognostic factor, but the risk score was. Higher infiltration of immune cells (B cells, CD4^+^ T cells, CD8^+^ T cells, neutrophils, macrophages, and dendritic cells) and higher levels of immune checkpoints (PD-1, CTLA-4, LAG-3, and TIM-3) were found in patients in the high-risk group. Finally, a novel nomogram model was constructed and evaluated to estimate the overall survival of LGG patients. In summary, our study provided new insights into immune infiltration in the tumor microenvironment and immunotherapies for LGGs.

## Introduction

Gliomas are the most common malignant tumors in the central nervous system (1). Traditionally, gliomas are divided into grades I to IV, including astrocytoma, oligoastrocytoma, oligodendroglioma, and glioblastoma (GBM) (2, 3). The phrase “low-grade glioma,” which refers to grade I and II gliomas, is being gradually replaced by term the “lower-grade glioma (LGGs).” Lower-grade gliomas comprise WHO grades II and III astrocytomas, oligodendrogliomas, and oligoastrocytomas (4, 5). Some LGGs tend to progress to WHO grade IV GBM within months, whereas others remain stable for years. The survival of patients with LGGs ranging from 1 to 15 years is closely related to therapeutic sensitivity (6). Because of high intraobserver and interobserver variability, the histopathological classification of LGGs has not been adequate for outcome prediction. As a result, genetic classification is also very important for guiding clinical decision-making (7). According to the updated classification system by the World Health Organization (WHO) in 2016, LGGs can be divided into three subtypes based on the mutation status of isocitrate dehydrogenase 1 (IDH1) and the codeletion status of 1p/19q, in which both tumor phenotypes and genotypes are considered (8). Although this classification system of LGGs has been adopted for molecular diagnosis, the known molecular markers are currently very limited for explaining the prognosis of LGGs. Thus, further exploration of the genetic mechanism and identification of new biomarkers to predict the prognosis of LGGs is important to develop precise treatments.

The tumor microenvironment plays an important role in tumor growth and development. Tumor-infiltrating immune cells are a critical part of the tumor microenvironment that regulate tumor growth and invasion. These immune cells include lymphocytes, natural killer (NK) cells, dendritic cells, macrophages, neutrophils, and so on (9). With the deep understanding of the tumor microenvironment, immunotherapy recently has been developed to be a more effective treatment for aggressive cancers (10–13). For example, multiple therapeutic antibodies that block immune checkpoints, such as cytotoxic T lymphocyte associated antigen 4 (CTLA4) and programmed cell death protein 1 (PD1), showed great effects in treating non-small-cell lung cancer, kidney cancer, and melanoma (14). In addition to solid tumors, immune checkpoint inhibitors (ICIs) have also shown remarkable efficacy in some refractory hematologic malignancies, such as leukemia and lymphoma (15). However, immunotherapy could only benefit a subset of current cancer patients, as some cancers are immunotherapy insensitive, some patients failed to respond at all, and some effective cases in the early stage achieved a limited response followed by tumor progression or recurrence (16). Thus, it is very important to find more immunotherapy targets and elucidate a more detailed molecular mechanism of immunotherapy responsiveness. Previous studies have shown that tumor mutation burden (TMB) has become a useful biomarker across many cancer types to predict the efficacy of immune checkpoint blockade (ICB) (16, 17). TMB is usually defined as the total number of somatic protein-coding base substitutions, but in some cases, it also includes insertion/deletion mutations. Theoretically, TMB should be determined by whole exome sequencing (WES) which is not routinely used as a clinical tool owing to its greater cost and complexity (17). With the development of next generation sequencing technology (NGS), large NGS-targeted panels are sufficient to substitute WES for TMB estimation in the clinic (17). Wang et al. found that TMB has a close relationship with immune infiltration and the prognosis of various cancers (18). However, few studies have focused on the relationship between TMB and immune infiltration in LGGs. Hence, we performed a comprehensive analysis to further explore the relationship between TMB and the immune response based on WES and RNA-sequencing (RNA-seq) data.

Currently, with the rapid development of sequencing technique, WES and RNA-seq data from many cancers are available in many public databases, such as The Cancer Genome Atlas (TCGA) database and Gene Expression Omnibus (GEO) database. In the present study, we downloaded somatic mutation and RNA-seq data of LGG patients from TCGA database. Then, we analyzed the influence of TMB on the immune microenvironment, and developed an immune-related risk score system based on six TMB-related immune genes to classify patients into high- and low-risk groups with distinct prognoses. Moreover, the risk score system was validated in the Chinese Glioma Genome Atlas (CGGA) dataset, and a reliable predictive nomogram model was constructed to evaluate overall survival (OS) for LGG patients. We believe that the immune-related risk score system has potential in patient management and that the selected hub genes can serve as potential therapeutic biomarkers for LGGs.

## Materials and Methods

### Somatic Mutation, RNA-seq Data, and Immune-Related Genes

The somatic mutation and RNA-seq expression data and corresponding clinical data sheets of LGGs were obtained from the TCGA database (https://cancergenome.nih.gov/) and used as the training dataset. The “maftools” R package was used to analyze and visualize the somatic mutation data (19). For RNA-seq data, only coding RNAs with an expression raw count value >10 in more than 75% of samples were retained for further analysis. We downloaded the RNA-seq data and corresponding clinical information from CGGA database (http://www.cgga.org.cn/) as the validation dataset. Furthermore, a comprehensive immune-related gene set was extracted from the Immunology Database and Analysis Portal (ImmPort) database (https://immport.niaid.nih.gov) (20).

### TMB Scores and Prognostic Analysis

In our study, the TMB score of each sample was calculated as the number of mutations/length of exons (30 Mb). Then, LGG samples were divided into high and low TMB groups according the median data and Kaplan–Meier analysis was conducted between the high and low TMB groups. Moreover, TMB levels were also assessed according to the WHO grade, histopathological types, and molecular subtype of LGGs.

### Differentially Expressed Genes (DEGs) and Gene Set Enrichment Analysis (GSEA)

Using the “limma,” “edgeR,” and “DEseq2” packages in the R software, the DEGs between the high and low TMB groups were obtained with the following thresholds: |Fold change| > 1 and False Discovery Rate (FDR) < 0.05. The expression profiles of genes were converted to log_2_(x+1) for further analysis. GSEA was performed between the high and low TMB groups using the JAVA8 platform. The reference gene set (c5.bp.v6.2.symbols.gm) was obtained from the MSigDB database (http://software.broadinstitute.org/gsea/msigdb/). Only enrichment pathways with a *p* < 0.05 and FDR < 0.25 were considered significant. In addition, the intersection between the DEGs and a list of 1,811 immune-related genes from the Immport database was selected for further analysis.

### Construction and Validation of Immune-Related Risk Score System

After excluding patients with missing mutation information and survival time <30 days, 474 samples were subjected to subsequent analysis. The clinical characteristics of these patients are shown in Table 1. Then, Kaplan–Meier analysis of the selected genes was performed to screen for prognostic genes in the TCGA dataset, which is then validated in the CGGA dataset. In total, six coding genes were significantly related to OS. To identify the best prognostic value of these genes, Cox analysis with least absolute shrinkage and selection operator (LASSO) L1-penalty was performed using the “glmnet” R package (21, 22). Finally, an immune-related risk score system was constructed utilizing Cox regression coefficients to multiply the expression values of immune genes in each patient. By applying the “survminer” R package, LGG patients were divided into low- and high-risk groups based on the optimal cutoff point of their risk score. Kaplan–Meier survival analysis and the log-rank test were employed to evaluate the prognostic value of this system. Using the “survival ROC” R package, we depicted time-dependent receiver operating characteristic (ROC) curves to evaluate the sensitivity and specificity of the system. The risk score system was also validated with CGGA database.

**Table 1:** Clinical characteristics of 474 LGG patients from TCGA cohort included in this study.

**Variables**	**Number (%)**
**Vital status**	
Alive	353 (74.47)
Dead	121 (25.53)
**Age**	
≤ 45	284 (59.92)
>45	190 (40.08)
**Gender**	
Female	214 (45.15)
Male	260 (54.85)
**Tumor grade**	
WHO II	229 (48.31)
WHO III	244 (51.48)
Unknown	1 (0.21)
**Histological type**	
Astrocytoma	176 (37.13)
Oligoastrocytoma	127 (26.79)
Oligodendroglioma	171 (36.08)
**IDH1 mutation and 1p/19 codeletion status**	
IDH1-mutant and 1p/19 codeletion	147 (31.01)
IDH1-mutant and 1p/19 non-codeletion	221 (46.62)
IDH1-wildtype	101 (21.31)
Others	5 (1.06)

*LGG, lower-grade glioma*.

### TIMER Database Analysis

The TIMER database (https://cistrome.shinyapps.io/timer) is a web tool for the comprehensive analysis and visualization of immune cells infiltration among 10,897 tumors from 32 cancer types (23). Six tumor-infiltrating immune subsets (B cells, CD4 T cells, CD8 T cells, macrophages, neutrophils, and dendritic cells) are included in TIMER. The abundance of the six immune cell types in the tumor microenvironment is estimated by a novel statistical method. Tumor immunological, clinical, and genomic features can be comprehensively explored in the TIMER database (23). In the “SCNA,” “Survival,” and “Gene” modules, the association between immune infiltration and somatic CNVs, clinical outcome, and gene expression, respectively, can be analyzed. Based on the three modules in the TIMER database, hub immune-related gene mutation types and the Kaplan–Meier analysis of immune infiltration cells were evaluated. Moreover, data on the immune infiltration levels of LGG samples were extracted from the TIMER database to calculate the correlation with the risk score system.

### Development and Evaluation of the Nomogram

To validate whether the risk score system has an independent prediction value, univariate and multivariate Cox regression analyses were performed together with traditional clinical features (gender, age, pathologic stage, IDH1 and 1p/19q status, and radiation status). Then, according to the results of multivariate Cox analysis using the “rms” R package, a nomogram was generated to predict the 1, 3, and 5-years survival probability. Calibrations and ROC analyses were used to predict the accuracy of the nomogram. A concordance index (C-index) was applied to evaluate the discrimination of the system.

### Statistical Analysis

Statistical analyses were conducted using the R software (version 3.5.1) and GraphPad Prism (version 7.0.0), and a *p* < 0.05 was considered statistically significant. The log-rank test was used in the Kaplan–Meier survival analysis. Student's *t*-test and Kruskal–Wallis test were employed in the two-group comparisons.

## Results

### Landscape of the LGG Mutation Profiles

**Figure 1:**
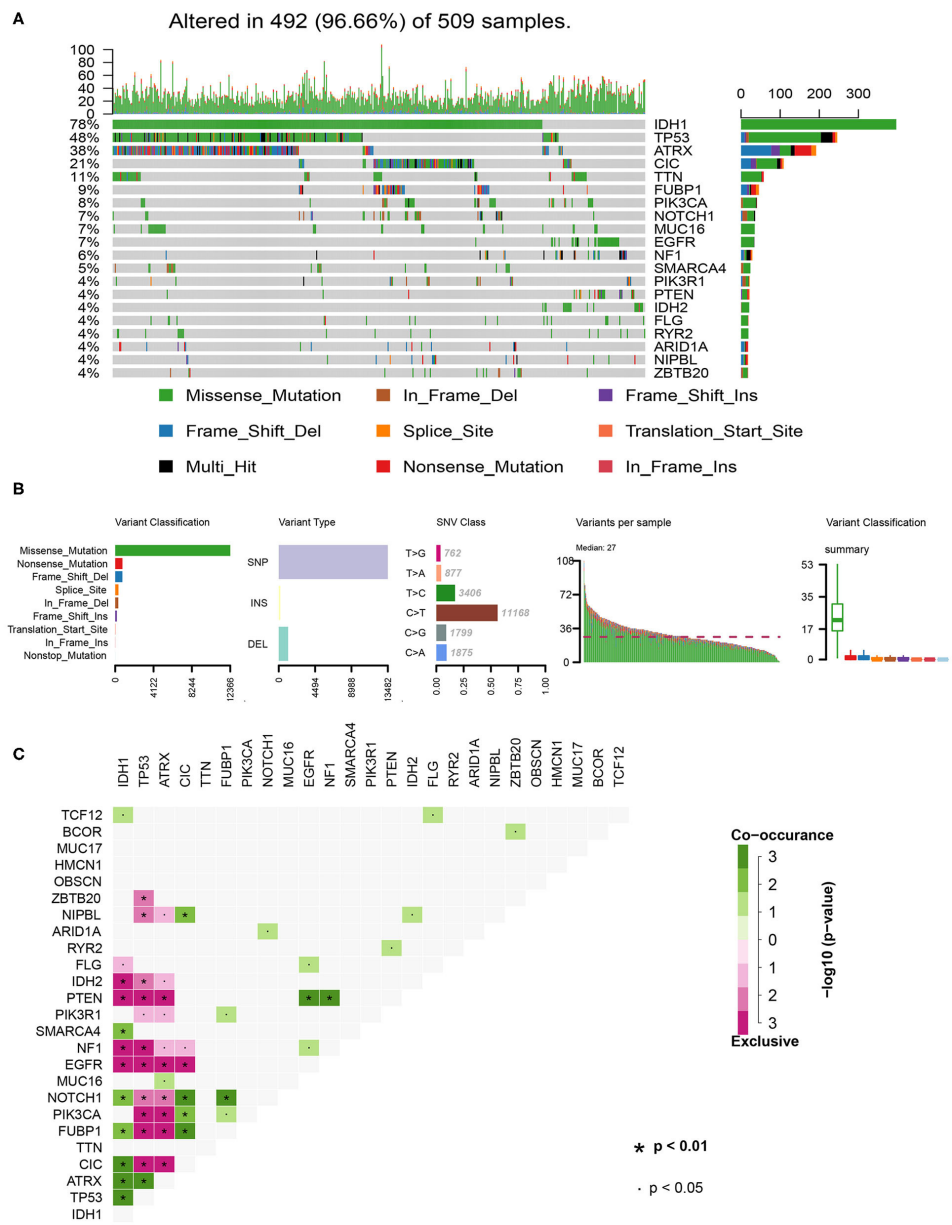
Summary of the of LGG mutation information. **(A)** Landscape of mutation profiles in LGG samples. Waterfall plot showing the mutation information for each gene. Different colors represented the different mutation types. **(B)** Classification of mutation types according to different categories and tumor mutation burden in specific samples. **(C)** The coincident and exclusive associations across the top 25 mutated genes. SNP, single nucleotide polymorphism; SNV, single nucleotide variant.

In total, we analyzed the somatic mutation profiles of 509 LGG patients in the VCF format using the “maftools” package. As shown in the waterfall plot, IDH1, TP53, and ATRX mutations are the top three mutated genes in LGG samples, and IDH1 mutations are found in more than 75% of LGG samples (Figure 1A). Moreover, missense mutations are the most common mutation classification, single nucleotide polymorphisms (SNPs) showed a higher fraction in the variant type than insertion or deletion, and C>T was the most common single nucleotide variant (SNV) in LGGs (Figure 1B). Furthermore, the number of variants in each sample was calculated, and the mutation types are also shown in box plot with different colors for LGGs (Figure 1B). The co-occurrence and exclusive associations between mutated genes are shown in Figure 1C.

### TMB Correlated With OS, WHO Grades, and Histopathological Types of LGGs

**Figure 2:**
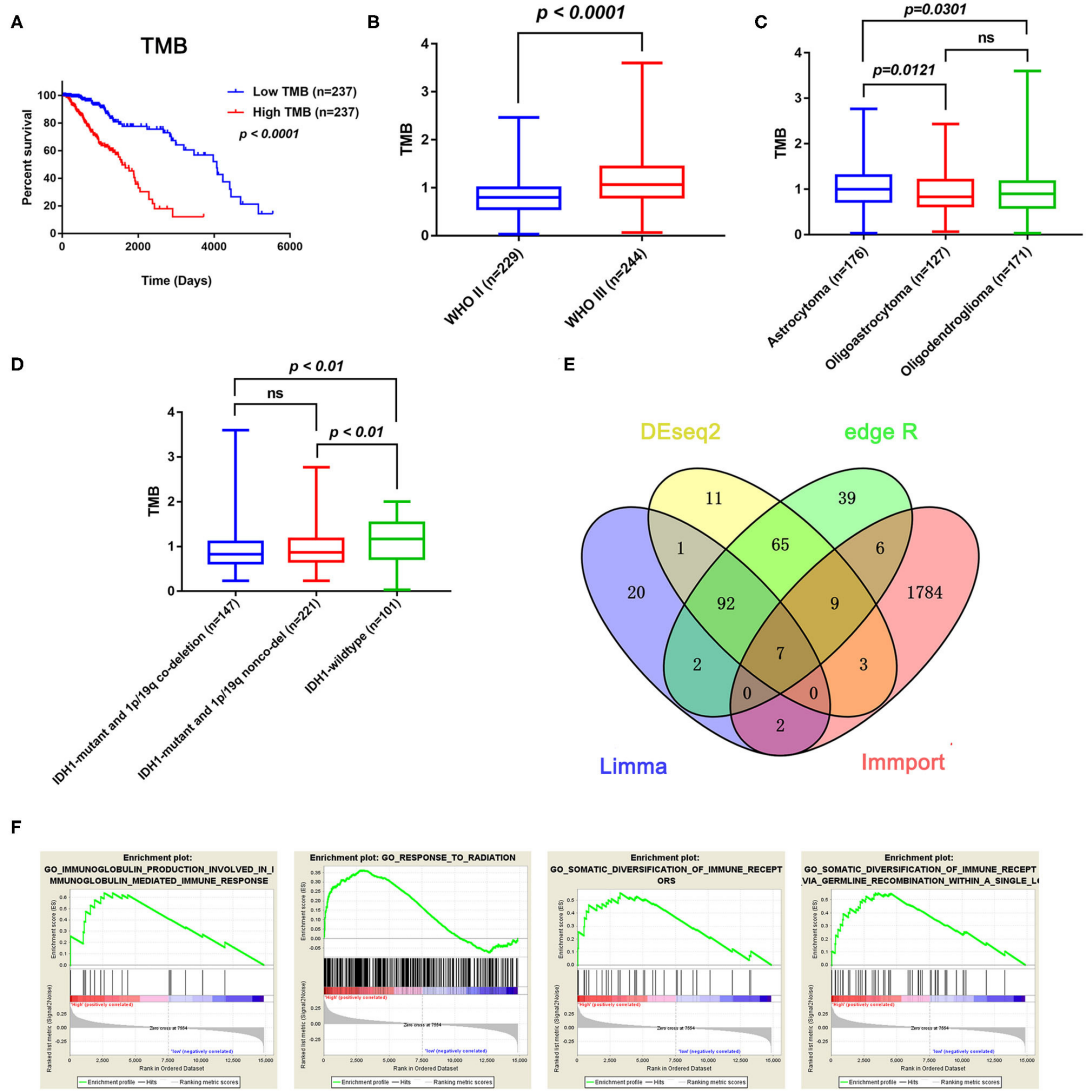
Prognostic value of TMB and a comparison of the gene expression profiles in high and low TMB groups. **(A)** Kaplan–Meier analysis showing that higher TMB levels correlated with a poor prognosis (*p* < 0.0001). **(B)** A higher TMB level was found in advanced grade tumors (*p* < 0.0001). **(C)** Comparison of the TMB levels with the different pathological types. Astrocytoma has higher TMB levels than oligoastrocytoma and oligodendroglioma (*p* = 0.0121 and *p* = 0.0301). **(D)** Lower TMB levels were associated with IDH1 mutant LGGs (*p* < 0.01). **(E)** Identification of TMB-related immune genes. **(F)** GSEA showed immune-related biological processes between the high- and low-risk groups.

After calculating the TMB value of each sample, all patients were divided into high and low TMB groups using the median TMB as the cutoff point. Interestingly, patients in high-TMB group have an obviously shorter OS than those in the low TMB group with *p* < 0.0001 (Figure 2A). We also found that the TMB levels are positively correlated with WHO grades (Figure 2B). Moreover, astrocytoma has higher TMB levels than oligoastrocytoma (*p* = 0.0121) and oligodendroglioma (*p* = 0.0301), but whereas TMB levels between oligoastrocytoma and oligodendroglioma showed no statistical significance (Figure 2C). Furthermore, LGG samples with an IDH1-mutant type have lower TMB levels than IDH1-wildtype samples; however, the TMB levels in IDH1-mutant and 1p/19q codeletion samples have shown no statistical significance when compared with IDH1-mutant and 1p/19q non-codeletion samples (Figure 2D).

### Identification of Immune-Related DEGs Between Low and High TMB Groups

In total, 14,848 coding genes were identified, according to the annotation information provided in the Ensembl database (http://asia.ensembl.org/index.html). Based on the limma, edgeR, and DEseq2 algorithms, 99 genes were identified as being differentially expressed between the low and high TMB groups, with thresholds of |Fold change| > 1 and FDR < 0.05. Among these genes, seven immune-related DEGs were identified by the ImmPort database for further analysis (Figure 2E). Moreover, GSEA analysis of the LGG samples in the high (*n* = 235) and low (*n* = 234) TMB groups was performed. The results showed that LGGs in the high TMB group were significantly enriched for 273 biological processes, and the following four immune-related biological processes were selected: GO_SOMATIC_DIVERSIFICATION_OF_IMMUNE_RECEPTORS (normalized enrichment score (NES) = 1.76, size = 56), GO_SOMATIC_DIVERSIFICATION_OF_IMMUNE_RECEPTORS_VIA_GERMLINE_RECOMBINATION_WITHIN_A_SINGLE_LOCUS (NES = 1.72, size = 50), GO_IMMUNOGLOBULIN_PRODUCTION_INVOLVED_IN_IMMUNOGLOBULIN_MEDIATED_IMMUNE_RESPONSE (NES = 1.60, size = 43), and GO_RESPONSE_TO_RADIATION (NES = 1.58, size = 385) (*p* < 0.05 and FDR < 0.25) (Figure 2F). In contrast, LGGs in the low TMB group did not enrich for any immune-related biological processes.

### Associations of Hub TMB-Related Immune Genes With Immune Infiltration

**Figure 3:**
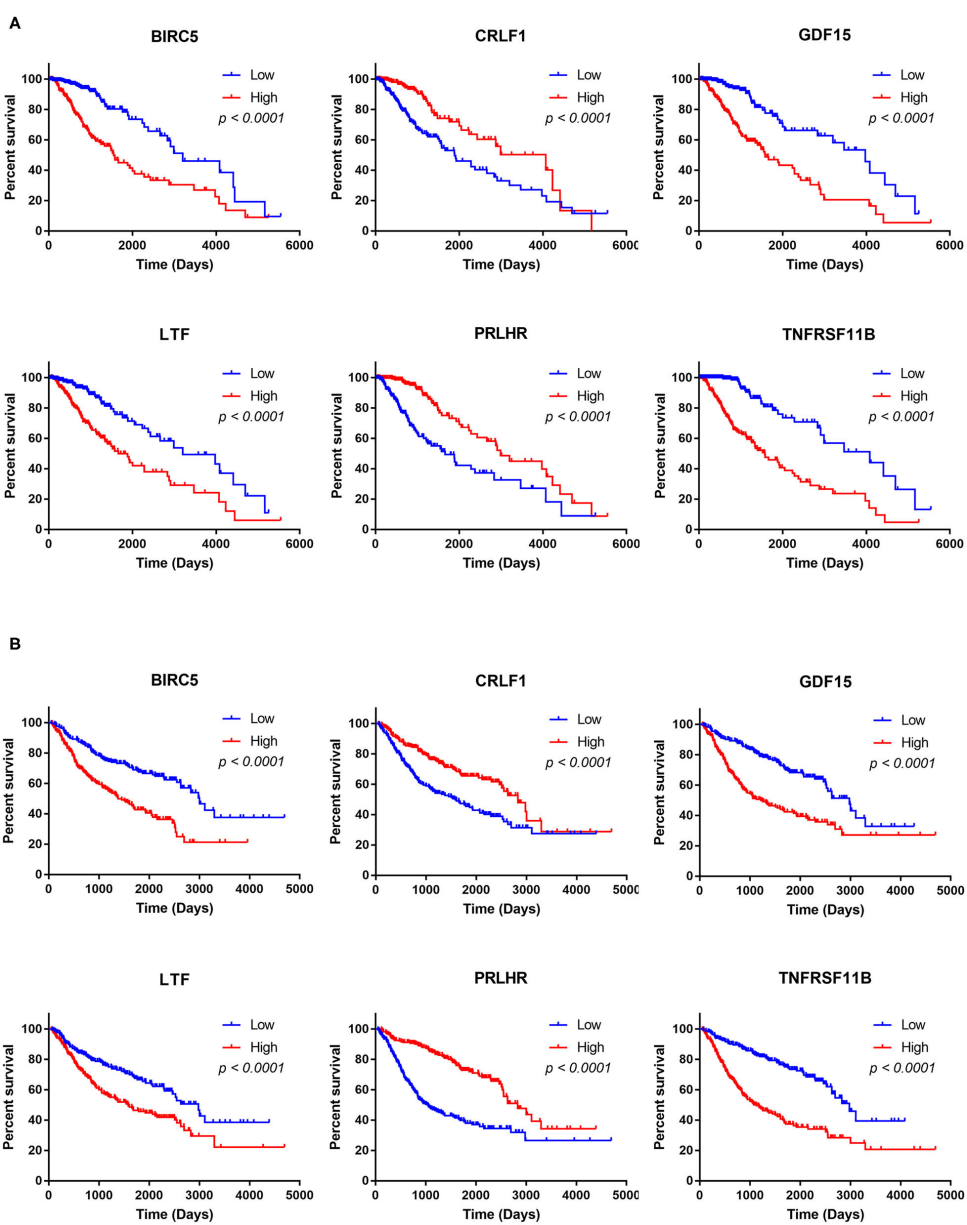
Kaplan–Meier analysis of the six hub TMB-related genes (BIRC5, CRLF1, GDF15, LTF, PRLHR, and TNFRSF11B) in TCGA database **(A)** and CGGA database **(B)**.

**Figure 4:**
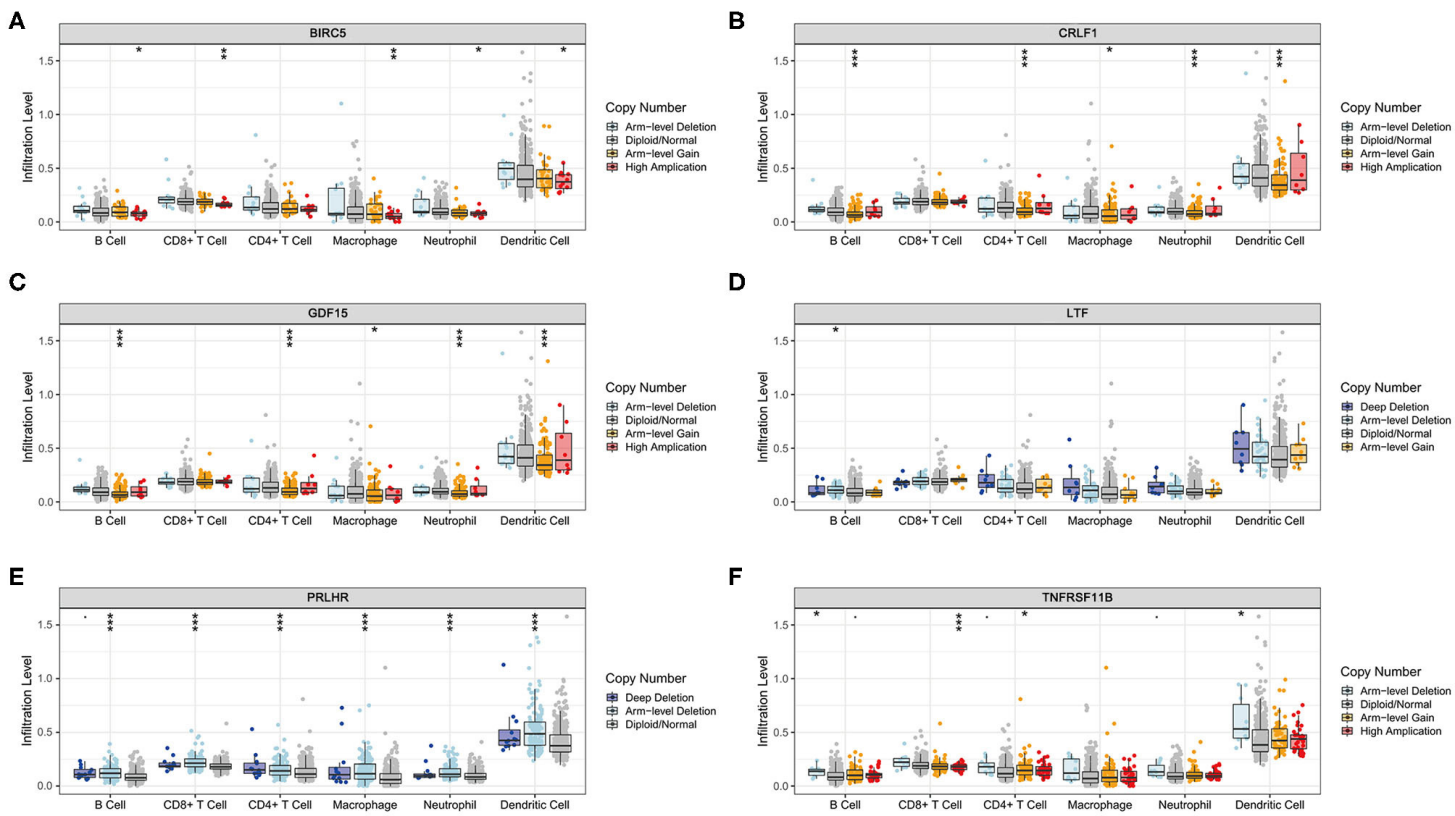
Immune cell infiltration levels of the six hub TMB-related gene mutants. **(A)** BIRC5, **(B)** CRLF1, **(C)** GDF15, **(D)** LTF, **(E)** PRLHR, **(F)** TNFRSF11B. *p < 0.05, **p < 0.01, and ***p < 0.001.

**Figure 5:**
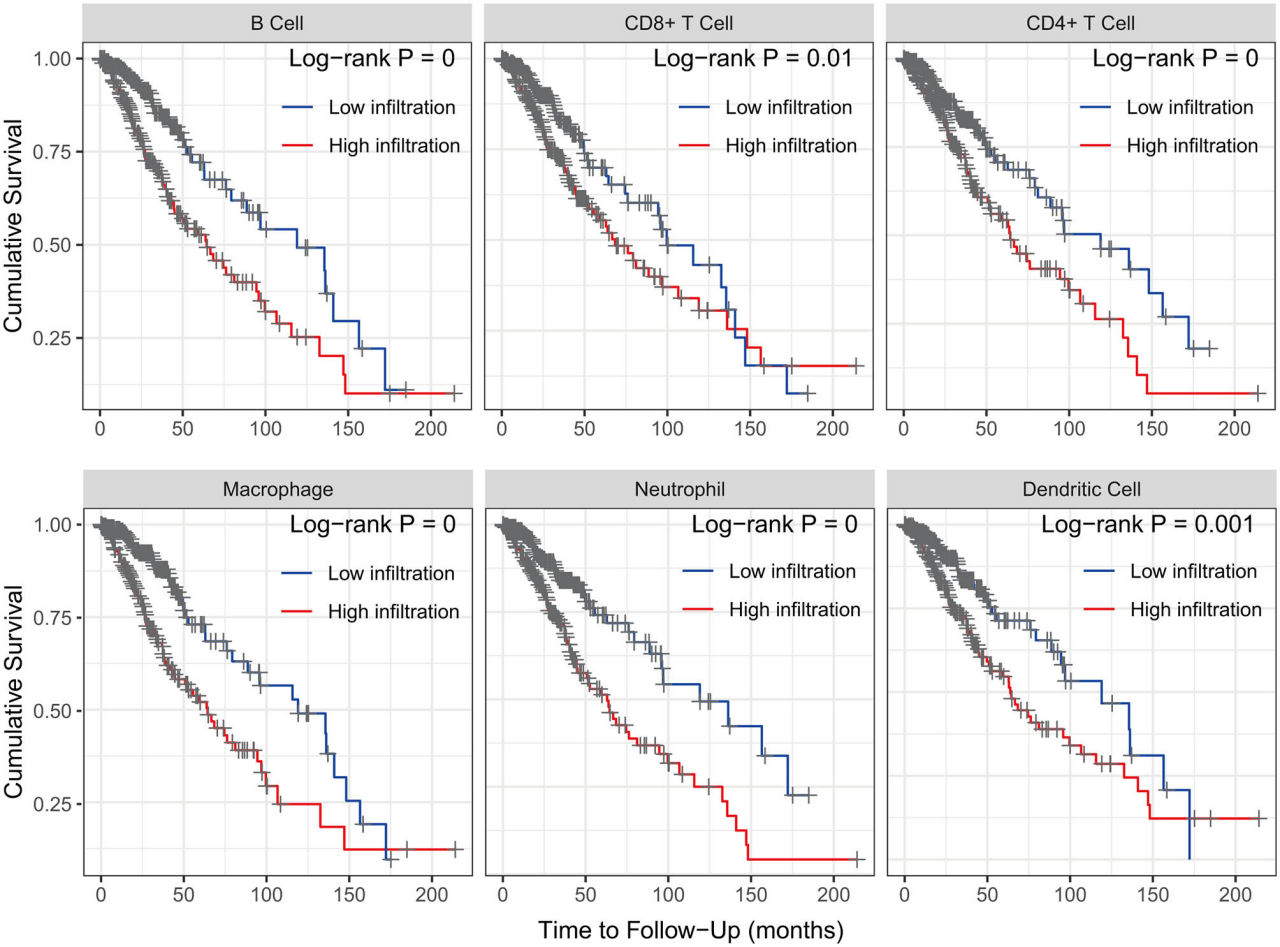
Kaplan–Meier analysis reveals that lower immune cell (B cells, CD4^+^ T cells, CD8^+^ T cells, neutrophils, macrophages, and dendritic cells) infiltration levels are correlated with poor survival outcomes in LGGs (*p* < 0.05).

**Figure 6:**
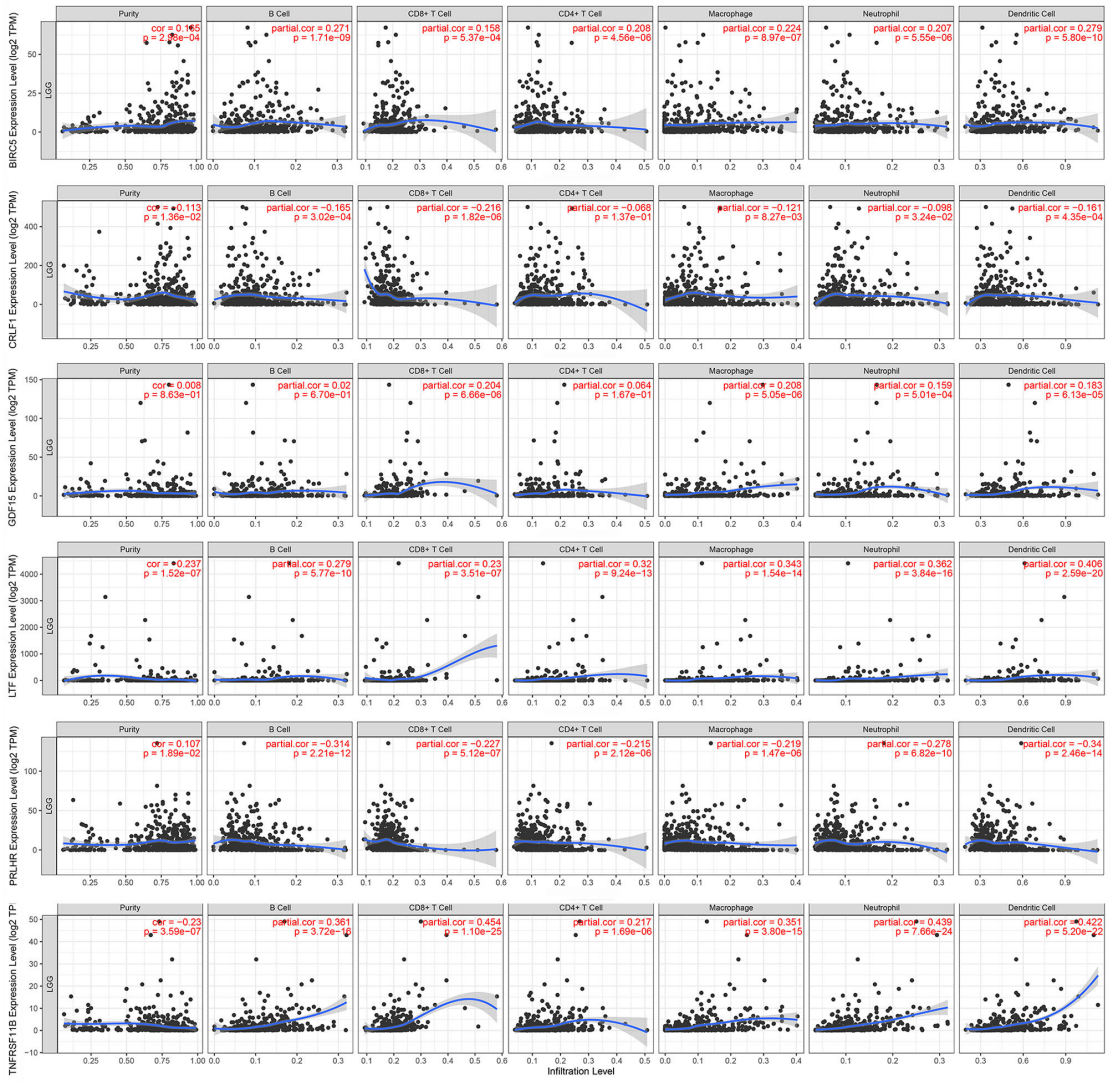
Correlation of hub gene expressions with immune infiltration levels in LGGs. The expression levels of BIRC5, GDF15, LTF, and TNFRSF11B were positively correlated with infiltrating levels of immune cells (B cell, CD8^+^ T cell, CD4^+^ T cell, macrophage, neutrophil, and dendritic cell). In contrast, the expression levels of CRLF1 and PRLHR were negatively correlated with infiltrating levels of immune cells (B cell, CD8^+^ T cell, CD4^+^ T cell, macrophage, neutrophil, and dendritic cell).

The Kaplan–Meier analysis results showed that six hub immune genes (BIRC5, CRLF1, GDF15, LTF, PRLHR, and TNFRSF11B) were highly associated with OS in LGGs. Higher expression levels of BIRC5, GDF15, LTF, and TNFRSF11B were positively correlated with poor prognosis, whereas higher expression levels of CRLF1 and PRLHR were negatively correlated with poor prognosis (Figure 3). Furthermore, we analyzed the underlying relationships between mutants of these hub genes with the immune infiltration microenvironment in LGGs based on the TIMER database. The results showed that mutants of these hub immune genes were related to the immune infiltration microenvironment in LGGs. Among them, mutants of BIRC5, CRLF1, and GDF15 inhibit the infiltration of several immune cells; in contrast, mutants of PRLHR promote the infiltration of several immune cells (Figure 4). Moreover, we found that the infiltration levels of immune cells (B cells, CD4^+^ T cells, CD8^+^ T cells, neutrophils, macrophages, and dendritic cells) was negatively correlated with the OS of patients in TCGA database (Figure 5). In addition, the correlation between the six hub genes and the level of immune cell infiltration was also analyzed in TIMER, and the results showed that the expression of BIRC5, GDF15, LTF, and TNFRSF11B were positively correlated with the infiltrating levels of immune cells, whereas the expression of CRLF1 and PRLHR were negatively correlated (Figure 6).

### Construction and Validation of the Immune-Related Risk Score System

**Figure 7:**
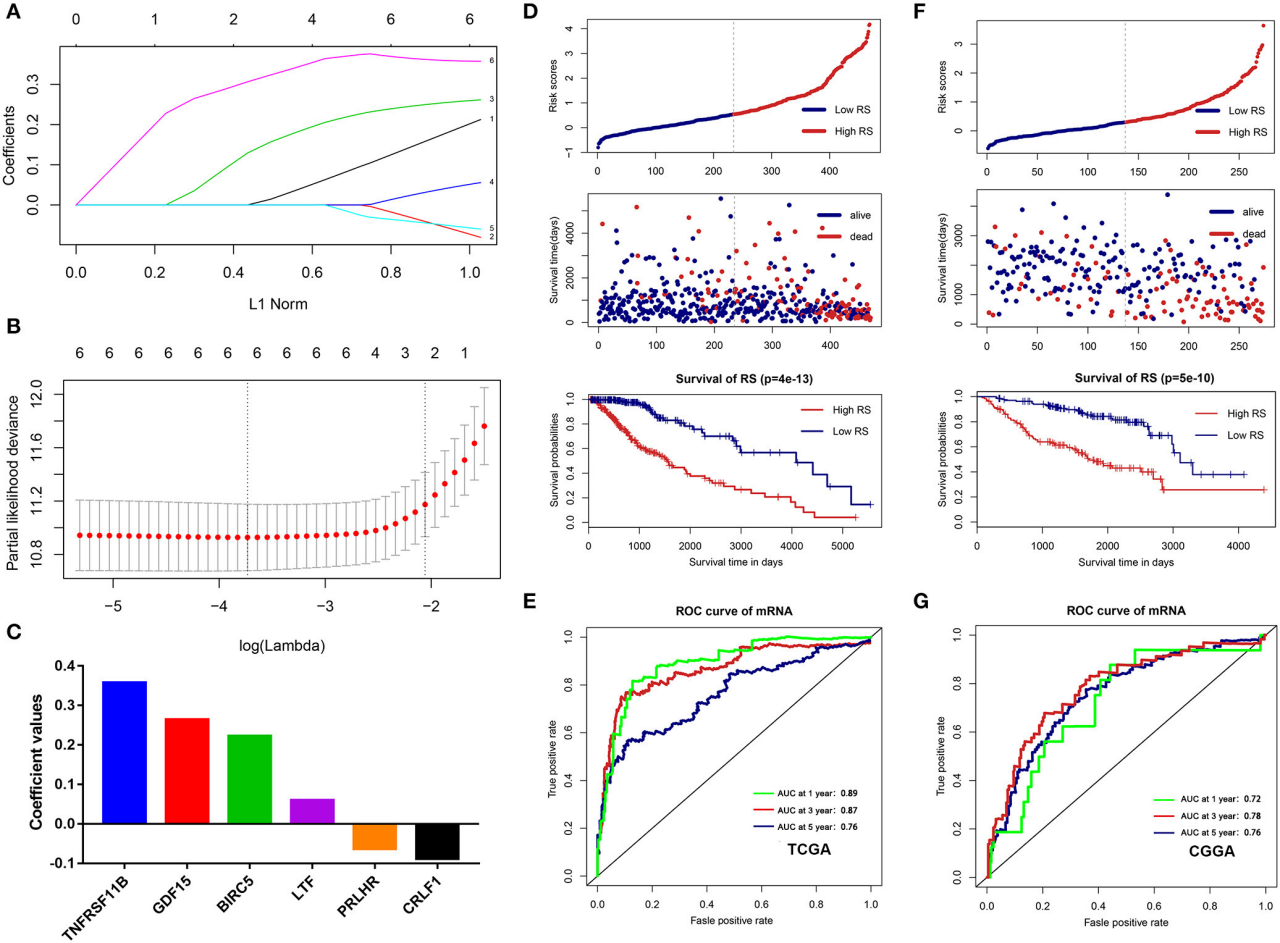
Construction and validation of the immune-related risk score system. **(A,B)** The six hub genes were all selected by LASSO Cox analysis in TCGA dataset. **(C)** Coefficient values for each gene. **(D)** Kaplan-Meier curves of the OS, risk scores distribution and survival status of each patient in the training cohort (TCGA dataset). **(E)** Time-dependent ROC curve analysis of the immune-related risk score system in the training cohort (TCGA dataset). **(F)** Kaplan-Meier curves of the OS, risk scores distribution and survival status of each patient in the validation cohort (CGGA dataset). **(G)** Time-dependent ROC curve analysis of the immune-related risk score system in the validation cohort (CGGA dataset).

To build an immune-related risk score system in the TCGA cohort, LASSO Cox analysis was performed to select genes (Figures 7A,B). Six hub immune genes were included in the immune-related risk score system. The risk scores formula is as follows: risk score = 0.222 × exp_BIRC5_ – 0.088 × exp_CRLF1_ + 0.264 × exp_GDF15_ + 0.060 × exp_LTF_ – 0.063 × exp_PRLHR_+ 0.357 × exp_TNFRSF11B_ (Figure 7C). Then, risk scores were calculated for each sample. Patients in the TCGA cohort were divided into high- and low-risk groups by the median value. The Kaplan–Meier analysis indicated that patients in the high-risk group have a poorer prognosis than patients in the low-risk group (*p* = 4e−13) (Figure 7D). Moreover, the time-dependent ROC curve analysis demonstrated a promising prognostic prediction (1-year AUC = 0.89, 3-years AUC = 0.87, 5-years AUC = 0.76) (Figure 7E).

To validate that the system had robust prognostic prediction ability, the same risk score formula was applied to the CGGA dataset. Using the median value of the risk score as a cutoff point, 274 LGG patients were divided into the high- and low-risk groups. Consistently, patients in the high-risk group had significantly poorer prognoses than patients in the low-risk group (Figure 7F). The ROC analysis also indicated that the system showed promising prognostic prediction (1-year AUC = 0.72, 3-years AUC = 0.78, 5-years AUC = 0.76) (Figure 7G).

### Immune Infiltration Landscape in the Low- and High-Risk Groups

**Figure 8:**
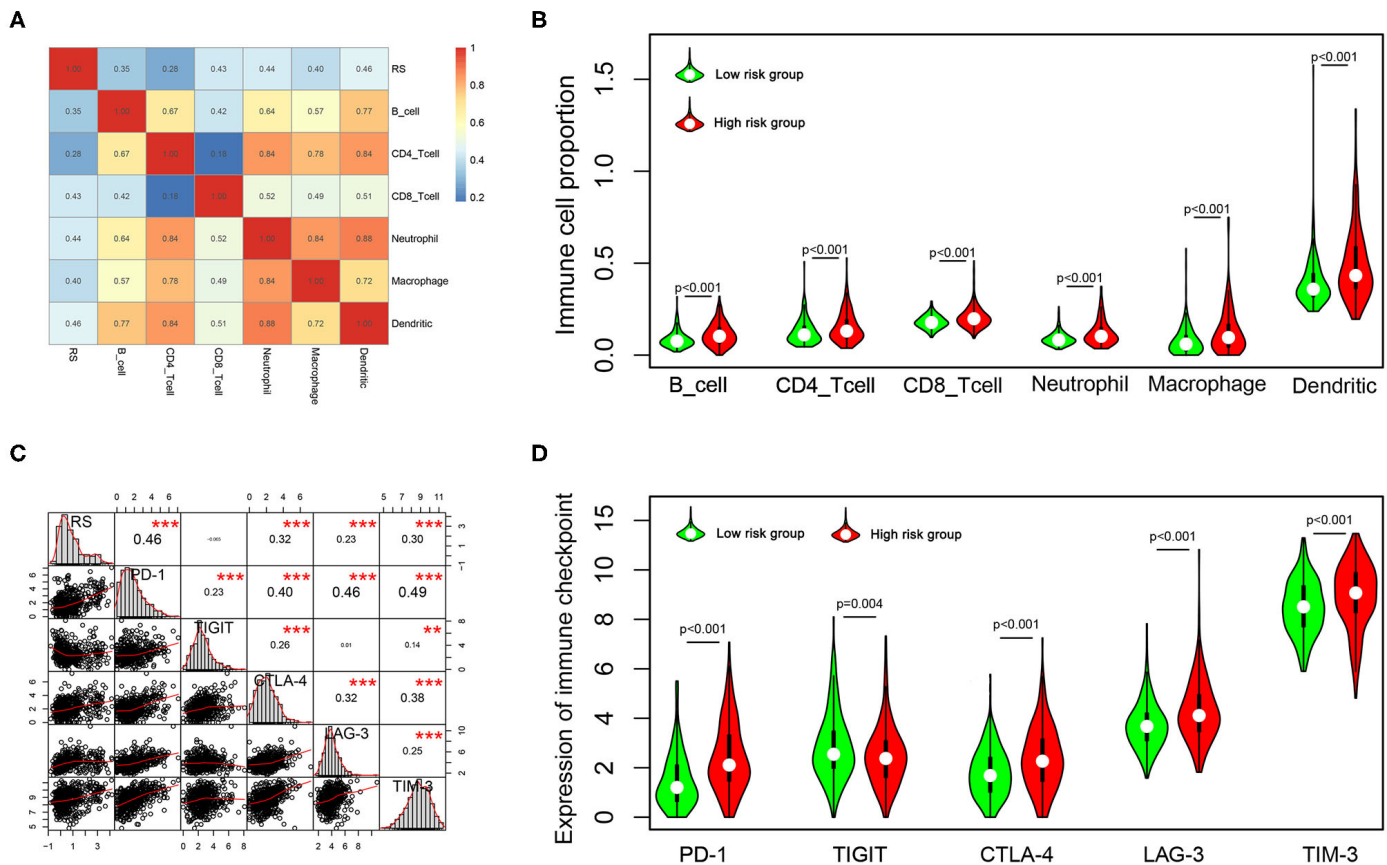
Correlation of the risk score with infiltrating immune cell proportions and immune checkpoints. **(A)** Correlation of the risk score with infiltrating immune cell proportions. Pearson's correlation coefficient values are shown in the heatmap. **(B)** Violin plot showing the immune cell proportions between low- and high-risk patients. **(C)** Correlation of the risk score with the expression of crucial immune checkpoints. Pearson's correlation coefficient values and level of significance were also showed. ***p < 0.001 and **p < 0.01. **(D)** The expression of different immune checkpoints between the low- and high-risk patients is shown in the violin plot.

To explore the potential relationship between our risk score system and the immune infiltration microenvironment, we analyzed the correlation between the risk score and infiltrating immune cells using the “TIMER” tool. The result showed that the risk score was positively correlated with infiltrating immune cells including B cells, CD4^+^ T cells, CD8^+^ T cells, neutrophils, macrophages, and dendritic cells (Figure 8A). Moreover, patients in the high-risk group had higher proportions of immune cell infiltration, with a *p* < 0.001 (Figure 8B).

Immune checkpoints play important roles in immune regulation, and their inhibitors are promising strategies for cancer treatment. Next, we explored the relationship between the risk score and the expression of critical immune checkpoints (PD-1, CTLA-4, LAG-3, TIM-3, and TIGIT). We found that the risk score showed a significantly positive correlation with PD-1, CTLA-4, LAG-3, and TIM-3 expression (Figure 8C). Further, we analyzed the expression of these immune checkpoints in the high- and low-risk groups, finding that patients in the high-risk group had higher expressions of PD-1, CTLA-4, LAG-3, and TIM-3 (*p* < 0.001), but lower expression of TIGIT (*p* = 0.001) (Figure 8D).

### Construction and Evaluation of the Nomogram Model

Next, univariate and multivariate Cox analyses were performed to comprehensively analyze whether the TMB and immune-related risk score system were independent prognosis factors for LGG patients. The univariate Cox analysis results showed that the TMB and risk score were significantly associated with OS. However, multivariate Cox analysis along with clinicopathological variables indicated that only the risk score can serve as an independent prognostic factor in the TCGA dataset (HR: 1.92, 95% CI: 1.92 (1.50–2.47), *p* = 2.33e−07; Table 2).

**Table 2:** Univariate and multivariate Cox regression analysis in TCGA.

**Characteristics**	**Univariate**		**Multivariate**	
	**HR (95% CI)**	***p*-value**	**HR (95% CI)**	***p*-value**
Age	1.06 (1.04–1.06)	**7.47e−14**	1.04 (0.01–4.66)	**3.14e−06**
Gender	0.98 (0.67–1.41)	0.9		
Grade	3.63 (2.37–5.55)	**2.99e−09**	1.50 (0.90–2.50)	0.12
IDH1-1P/19q status	0.18 (0.12–0.27)	**5.5e−14**	0.68 (0.48–0.97)	**0.03**
Radiation	2.23 (1.41–3.53)	**6.4e−04**	1.06 (0.65–1.74)	0.80
TMB	3.51 (2.46–5.01)	**3.59e−12**	1.21 (0.74–1.95)	0.45
RS	2.73 (2.30–3.23)	**2.71e−31**	1.92 (1.50–2.47)	**2.33e−07**

*Gender was defined as 1, female; 2, male; Grade was defined as 1, G2; 2, G3; IDH1-1P/19q status was given a value of 0, IDH1-wildtype; 1, IDH1-mutant and 1p/19q non-codeletion; 2, IDH1-mutant and 1p/19q codeletion. Bold values represent statistical significance (p < 0.05)*.

**Figure 9:**
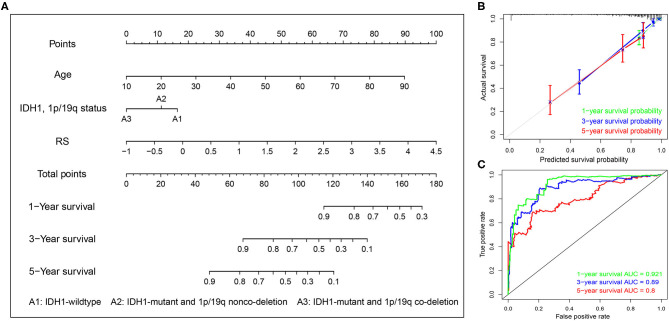
Construction of the nomogram based on TCGA dataset. **(A)** A nomogram for the quantitative prediction of 1-, 2-, and 3-years survival for LGG patients based on the RS, age and IDH1 and 1p/19q status. **(B)** Calibration curves for the nomogram. **(C)** Time-dependent ROC curves for the 1-, 2-, and 3-years survival used to assess the nomogram model.

To systematically predict the prognosis of LGGs, we constructed a nomogram model based on the risk score and two independent prognostic factors (age and IDH1 and 1p/19q status) in the TCGA dataset (Figure 9A). The C-index for the nomogram was 0.862 indicating a high discrimination ability, and calibration plot showed excellent concordance for the 1-, 3-, and 5-years predicted and actual OS probabilities (Figure 9B). Moreover, the ROC curve analysis also demonstrated a satisfactory prediction for sensitivity and specificity with a 1-year AUC of 0.921, 3-years AUC of 0.89, and 5-years AUC of 0.80 (Figure 9C).

## Discussion

Genomic variations are considered a major cause of LGGs (24). Although great efforts have been made in neurosurgery, radiotherapy, and chemotherapy, the survival of LGG patients still ranges widely. Recently, immunotherapy has shown promising results in the treatment of advanced or aggressive cancers (25). Although many efforts have been made for glioma immunotherapy, there is still a lack of reliable molecular biomarkers to distinguish patients with potential sensitivity to immunotherapy (26). Hence, it is particularly important to identify more immune-related prognostic biomarkers that can be potential therapeutic targets or can be used to screen immunotherapy-sensitive patients.

TMB is a novel biomarker to predict cancer immunotherapeutic response, which has been shown to be effective for many tumors, such as lung cancer (11), melanoma (27), and so on. Wang et al. found that a high TMB may promote cancer-testis antigen expression and inflammatory response, and patients with a higher TMB could gain a more favorable prognosis if treated with immunotherapy in a variety of cancers (18). However, few studies have focused on the prognostic role of TMB and the association between TMB and immune cell infiltration in LGGs. Thus, we aimed to explore the prognostic role of TMB and its potential association with immune infiltration in LGGs in this study.

Interestingly, the results of the Kaplan–Meier analysis showed that patients in high TMB group had a poorer prognosis, higher tumor grades, and advanced pathological subtypes. A recent bioinformatics study showed that the high tumor proliferative activity in high TMB patients may lead to a shorter OS of glioma, but more experiments are still needed to validate their findings (28). As high TMB patients usually benefit from immunotherapy (17, 29), higher TMB glioma patients may be able to achieve a better prognosis once immunotherapy is widely utilized in the treatment of glioma. Moreover, GSEA analysis showed that more immune-related biological processes were enriched in the high TMB group, indicating that a high TMB enhanced the immune phenotype. Then, we identified six hub immune genes that were highly associated with OS in LGGs. Among them, the expression levels of four genes (BIRC5, GDF15, LTF, and TNFRSF11B) were negatively correlated with OS, whereas two genes (CRLF1 and PRLHR) were positively correlated with OS. Analysis with the TIMER database showed that high immune cells (B cells, CD4^+^ T cells, CD8^+^ T cells, neutrophils, macrophages, and dendritic cells) infiltration was proven to be associated with a poorer prognosis in LGGs. Indeed, the expression levels of BIRC5, GDF15, LTF, and TNFRSF11B were positively correlated with infiltration levels of immune cells, whereas the expression levels of CRLF1 and PRLHR were negatively correlated. Furthermore, TIMER database analysis also showed that the mutants of these hub immune genes were correlated with immune infiltration in LGGs.

BIRC5 (survivin), a member of the inhibitor of apoptosis proteins family, can suppress cell apoptosis and regulate cell proliferation. BIRC5 is overexpressed in various tumors and has been found as a prognostic marker in gastric cancer (30), renal cell carcinoma (31), and breast cancer (32). However, the function of BIRC5 in LGGs has never been reported. GDF15 is a member of the growth differentiation factors (GDFs) subfamily that belongs to transforming growth factor beta superfamily (33). Peng et al. found that GDF15 might be able to regulate the expression of PD-L1, and targeting the GDF15/PD-L1 pathway might be promising for the treatment of GBM patients (33). The lactoferrin (LTF) gene, an iron-binding protein that is involved in innate and adaptive immunity, acts as a tumor suppressor gene in several tumors, including nasopharyngeal carcinoma (34), prostate carcinogenesis (35), and so on. In contrast, it appears to be a cancer-promoting factor in LGGs, even though further studies are needed to clarify the underling mechanism. TNFRSF11B is a cytokine receptor and belongs to the tumor necrosis factor (TNF) receptor superfamily. Deng et al. demonstrated that TNFRSF11B was a prognostic biomarker and related to worse survival in LGGs for the first time (36). CRLF1, cytokine receptor-like factor 1, stimulates neuronal growth and differentiation and has been proven to be involved in neuroprotection (37). However, its function in LGGs remains unclear and our study found that elevated CRLF1 expression was related to worse survival in LGGs. PRLHR (prolactin releasing hormone receptor), namely G-protein-coupled receptor 10, is the receptor for prolactin releasing peptide (PrRP). Previous studies found that it was associated with the regulation of feeding and energy balance (38). Su et al. found that PRLHR gene variants are protective factors in colorectal cancer patients of Chinese Han population (39). More studies are needed to explore the function of the PRHLR gene in LGGs.

In this study, we comprehensively studied the role of TMB in the regulation of immune phenotype in LGGs. Then, an immune-related risk score system was developed based on the TCGA dataset and validated with the CGGA dataset. This risk score system has favorable prognostic prediction ability, which is independent of traditional prognostic factors, such as IDH1 and 1p/19q status, age, and WHO grade. More importantly, we constructed a novel nomogram model integrated risk score with age and IDH1 and 1p/19q status to predict the OS of LGG patients. According to the risk score system and nomogram, clinicians can calculate an individual score for a patient and then can predict the 1-, 3-, and 5-years OS. Using the TIMER database, we found that higher infiltrating levels of B cells, CD4^+^ T cells, CD8^+^ T cells, neutrophils, macrophages, and dendritic cells are all negatively correlated with the OS of LGGs. Furthermore, we explored the correlation between the risk score and immune cell infiltration in LGGs. The results showed that patients in the high-risk group had higher infiltrating levels of B cells, CD4^+^ T cells, CD8^+^ T cells, neutrophils, macrophages, and dendritic cells than patients in the low-risk group. This indicated that higher levels of immune cell infiltration in the high-risk group may contribute to the poorer prognosis. These results also suggested that immune cell infiltration plays a crucial role in the progression of LGGs. Thus, the risk score system could be used as a predictor for prognosis and immune cell infiltration, and has good prospects for clinical application.

Currently, many clinical trials are evaluating the effect of ICIs in glioma (40). Furthermore, we analyzed the association between the risk score and the expression of critical immune checkpoints. The result showed that patients in high-risk group had higher PD-1, CTLA-4, LAG-3, and TIM-3 expression. The immunosuppressive microenvironment may lead to a poor prognosis in these patients. Thus, the patients in the high-risk group were more likely to benefit from ICIs.

Although a previous study had developed an IDH1-associated immune prognostic signature for LGGs (36), no study has systematically explored the relationships between TMB and immune infiltration and constructed a TMB-related risk score system in LGGs. Thus, our study provides new insights into the immune cell infiltration of tumor microenvironment and immunotherapies for LGGs. However, there were some limitations to our study. First, this is a retrospective study, and the results should be further confirmed by prospective studies. Moreover, more experiments are needed to elucidate the underlying mechanisms of the selected genes on immune cell infiltration in the future.

In conclusion, TMB was negatively correlated with OS and a high TMB might inhibit immune infiltration in LGGs. The TMB-related immune-related risk score system can divide patients into low- and high-risk groups with different outcomes and immunophenotypes. Moreover, the patients in the high-risk group are more likely to benefit from ICI treatment in the future. These findings may aid clinicians in identifying patients who are most likely to benefit from ICIs and to develop valuable personalized immunotherapy regimens for LGG patients in the future.

## Data Availability Statement

The datasets for this study can be found in TCGA (http://cancergenome.nih.gov/) and CGGA (http://www.cgga.org.cn/).

## Author Contributions

XJ, CR, and GT conceived and designed the study. WY wrote the manuscript. JT, ZX, QZ, CZ, and XF analyzed the results. ZW, YG, and ZJ performed the image visualization. All the authors approved the final manuscript.

## Conflict of Interest

The authors declare that the research was conducted in the absence of any commercial or financial relationships that could be construed as a potential conflict of interest.
